# Supplementary material for: A Humanized Yeast Phenomic Model of Deoxycytidine Kinase to Predict Genetic Buffering of Nucleoside Analog Cytotoxicity
Source: Genes (Basel). 2019 Sep 30;10(10):770. doi: 10.3390/genes10100770 (PMC6826991; doi:10.3390/genes10100770)
Supplement: Supplementary file 1 [file genes-10-00770-s001.zip › Additional_Files/Additional_File7_GOTermHeatmaps.zip!/Additional_File7_GOTermHeatmaps/transcription,_DNA-templated.pdf]

transcription, DNA−templated

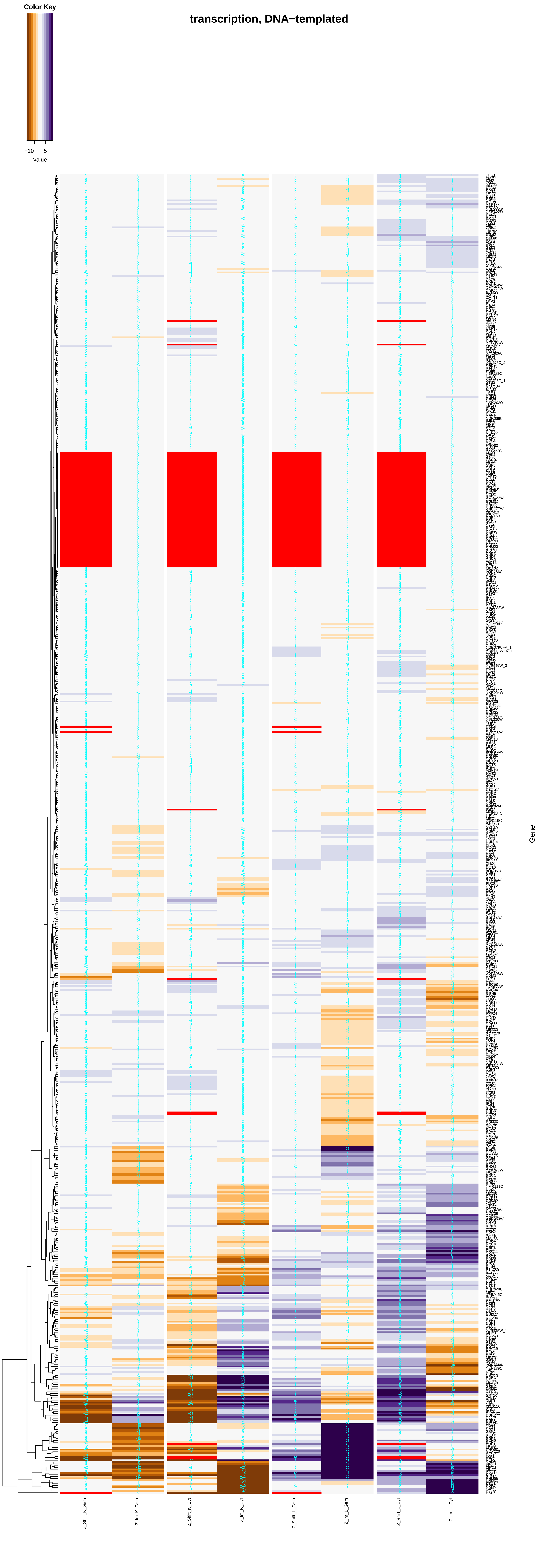

transcription from RNA polymerase I promoter

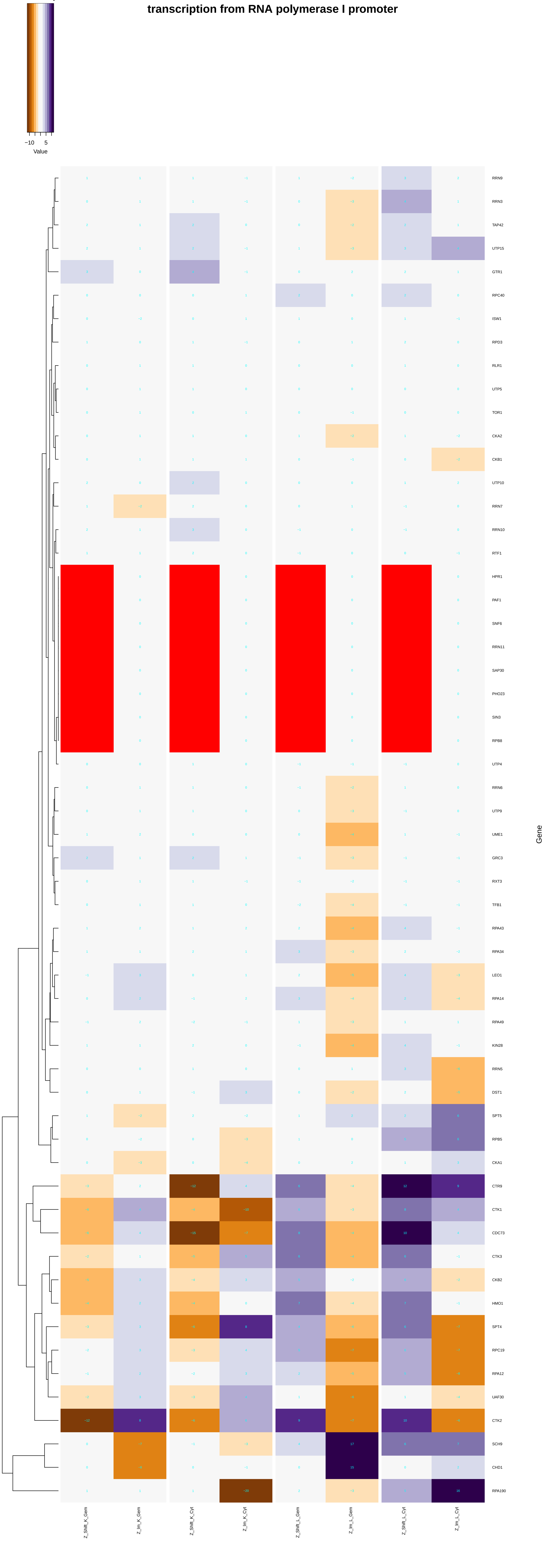

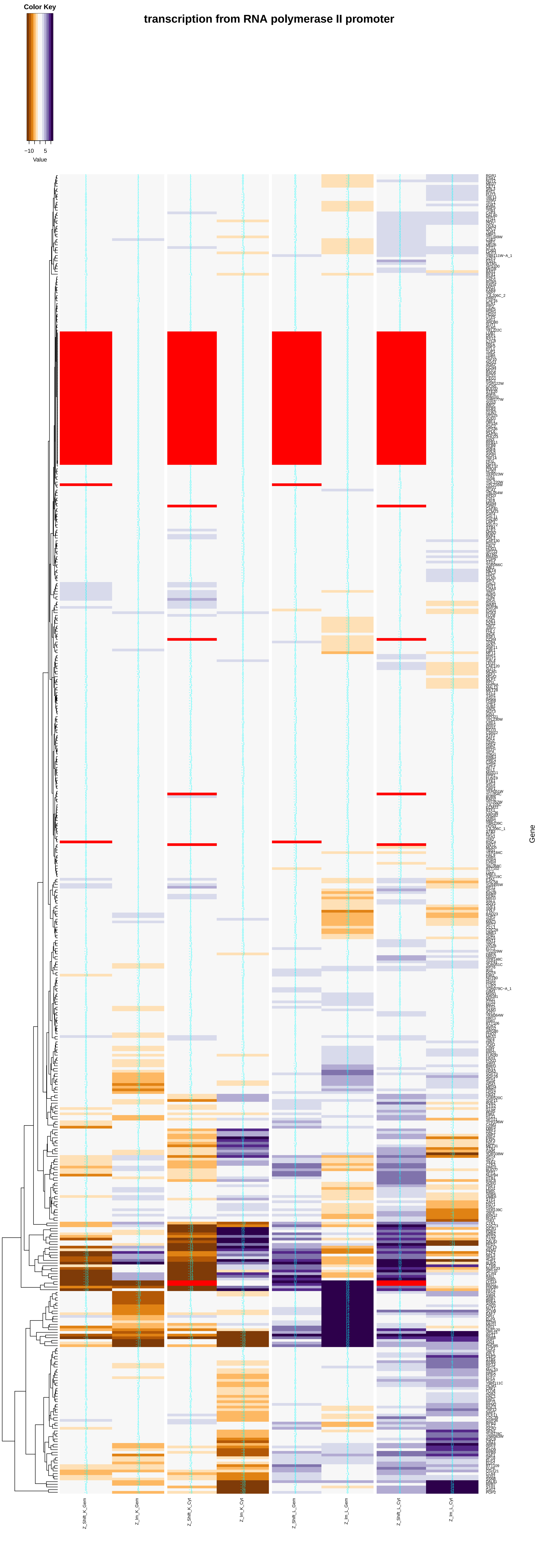
transcription from RNA polymerase II promoter
Color Key
−10
5
Value
Gene
Z_Shift_K_Gem
Z_Im_K_Gem
Z_Shift_K_Cyt
Z_Im_K_Cyt
Z_Shift_L_Gem
Z_Im_L_Gem
Z_Shift_L_Cyt
Z_Im_L_Cyt

transcription from RNA polymerase III promoter

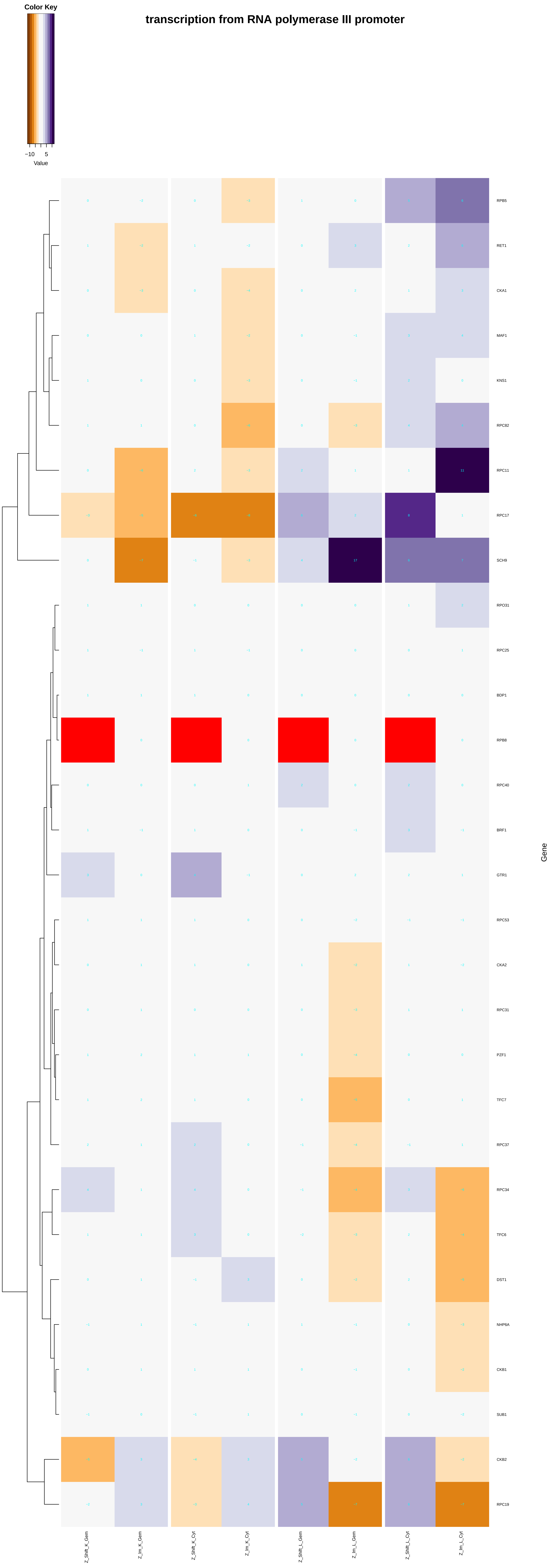

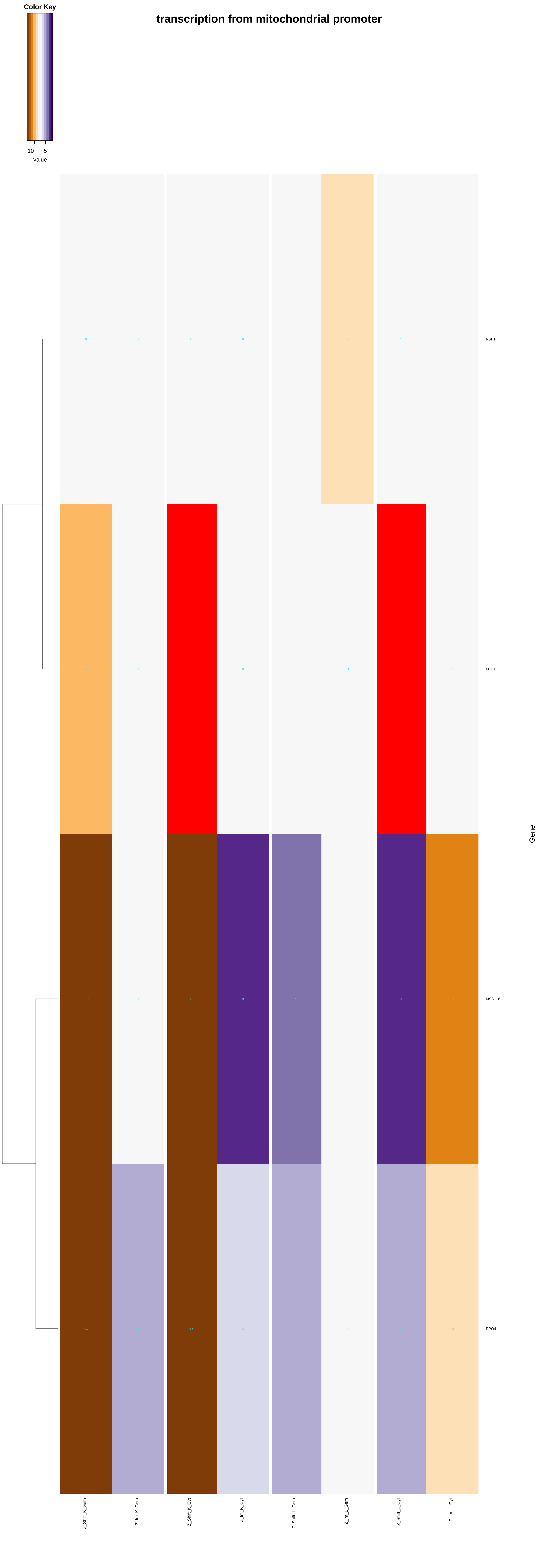
transcription from mitochondrial promoter
Color Key
−10
5
Value
RSF1
MTF1
MSS116
RPO41
Gene
Z_Shift_K_Gem
Z_Im_K_Gem
Z_Shift_K_Cyt
Z_Im_K_Cyt
Z_Shift_L_Gem
Z_Im_L_Gem
Z_Shift_L_Cyt
Z_Im_L_Cyt

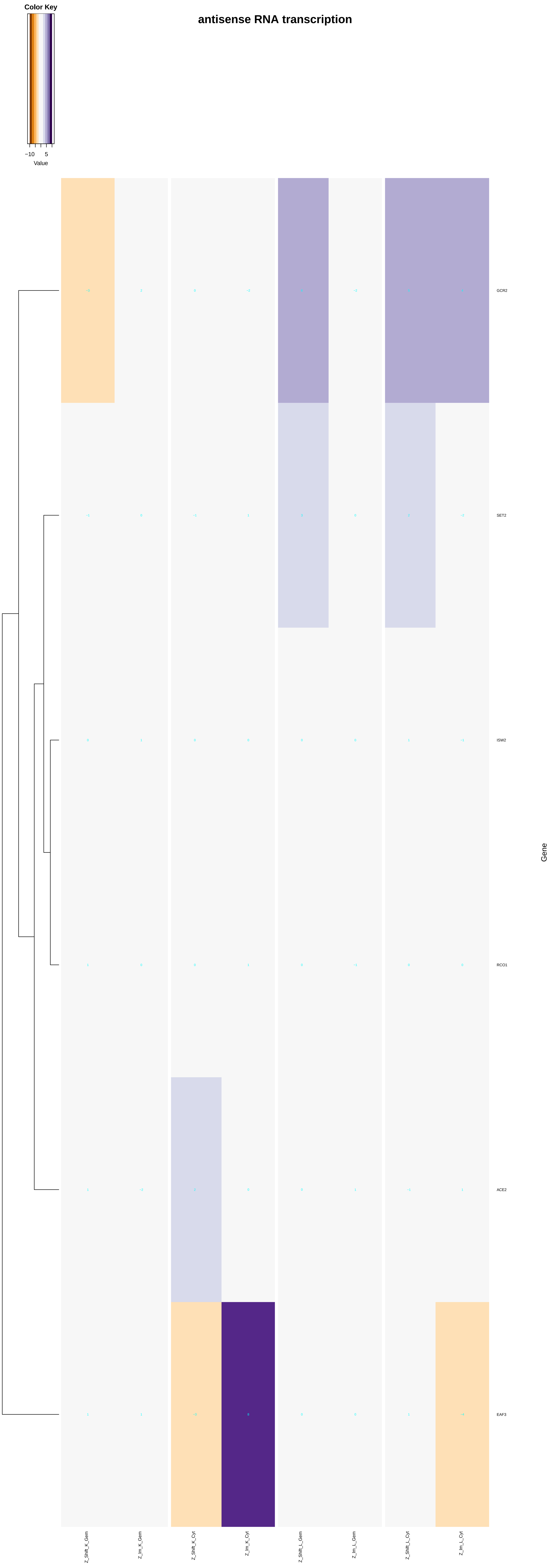
antisense RNA transcription
Color Key
−10
5
Value
GCR2
SET2
ISW2
RCO1
ACE2
EAF3
Gene
Z_Shift_K_Gem
Z_Im_K_Gem
Z_Shift_K_Cyt
Z_Im_K_Cyt
Z_Shift_L_Gem
Z_Im_L_Gem
Z_Shift_L_Cyt
Z_Im_L_Cyt

ncRNA transcription

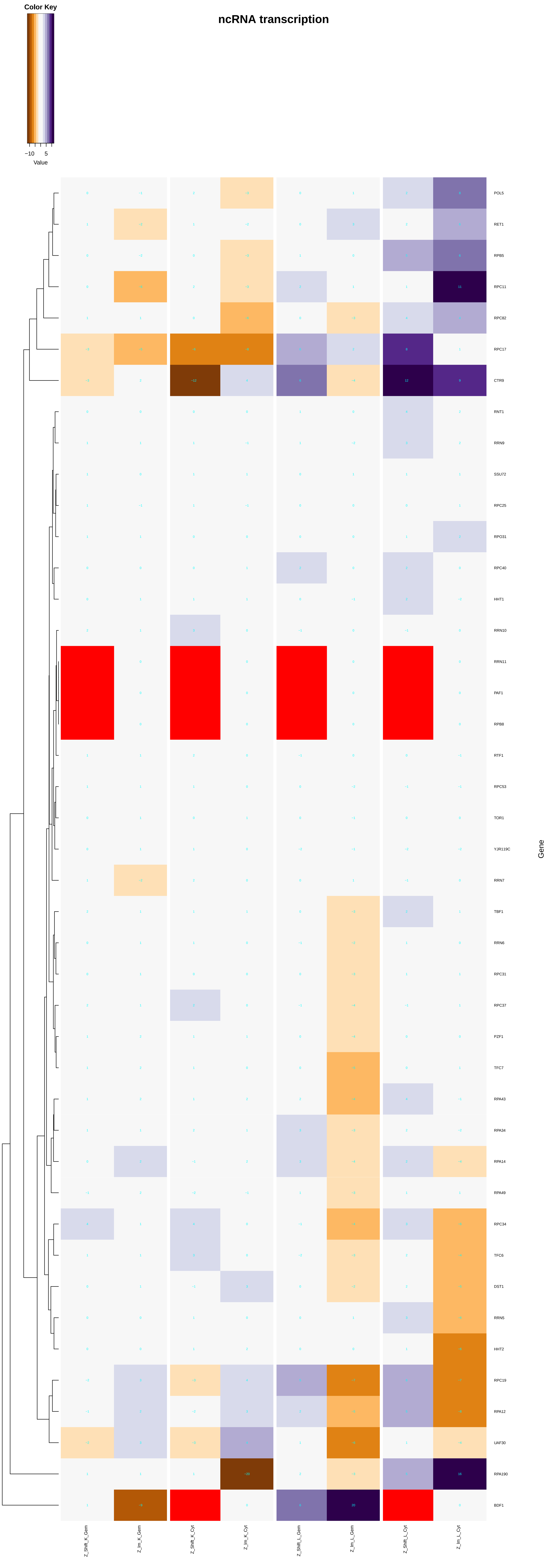

transcription of nuclear large rRNA transcript from RNA polymerase I promoter

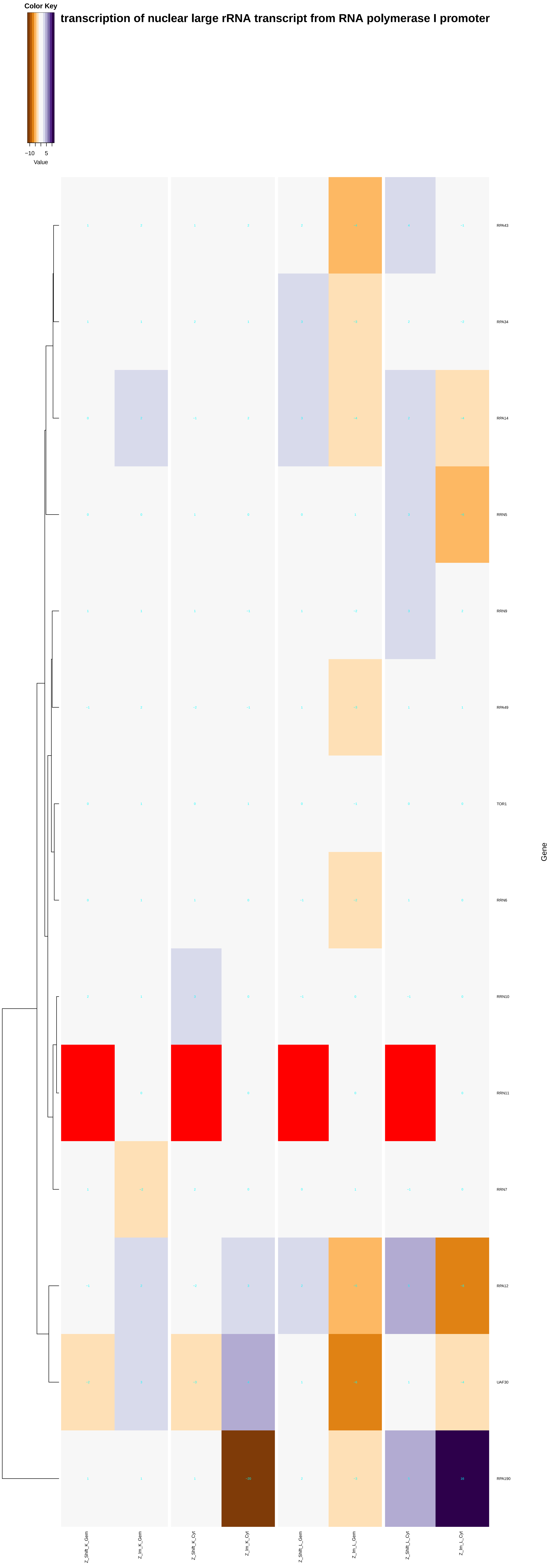

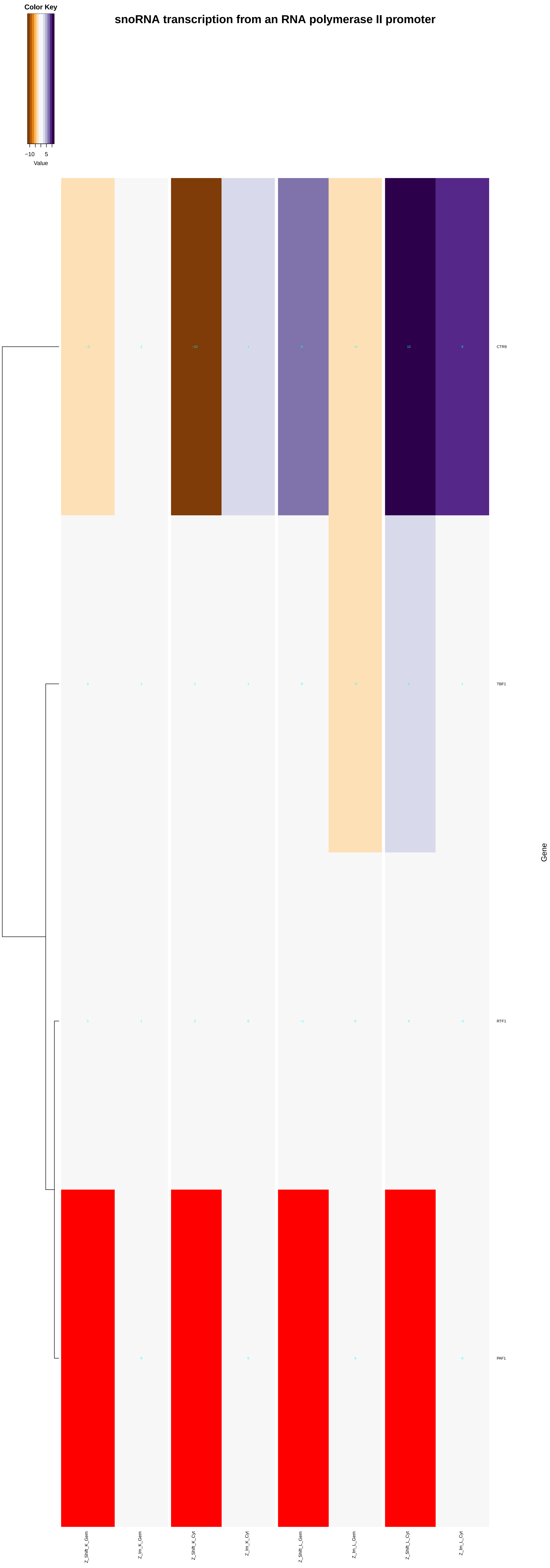

snoRNA transcription from an RNA polymerase II promoter
Color Key
−10
5
Value
CTR9
TBF1
RTF1
PAF1
Gene
Z_Shift_K_Gem
Z_Im_K_Gem
Z_Shift_K_Cyt
Z_Im_K_Cyt
Z_Shift_L_Gem
Z_Im_L_Gem
Z_Shift_L_Cyt
Z_Im_L_Cyt

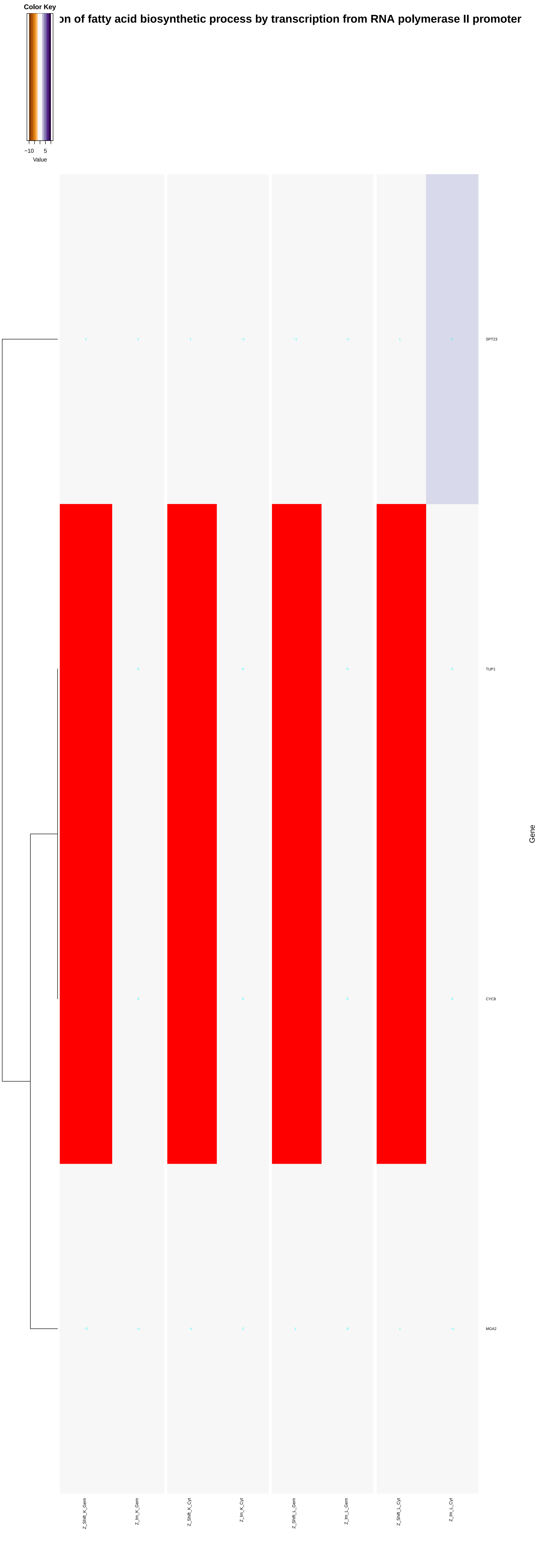

on of fatty acid biosynthetic process by transcription from RNA polymerase II promoter
Color Key
−10
5
Value
SPT23
TUP1
CYC8
MGA2
Gene
Z_Shift_K_Gem
Z_Im_K_Gem
Z_Shift_K_Cyt
Z_Im_K_Cyt
Z_Shift_L_Gem
Z_Im_L_Gem
Z_Shift_L_Cyt
Z_Im_L_Cyt

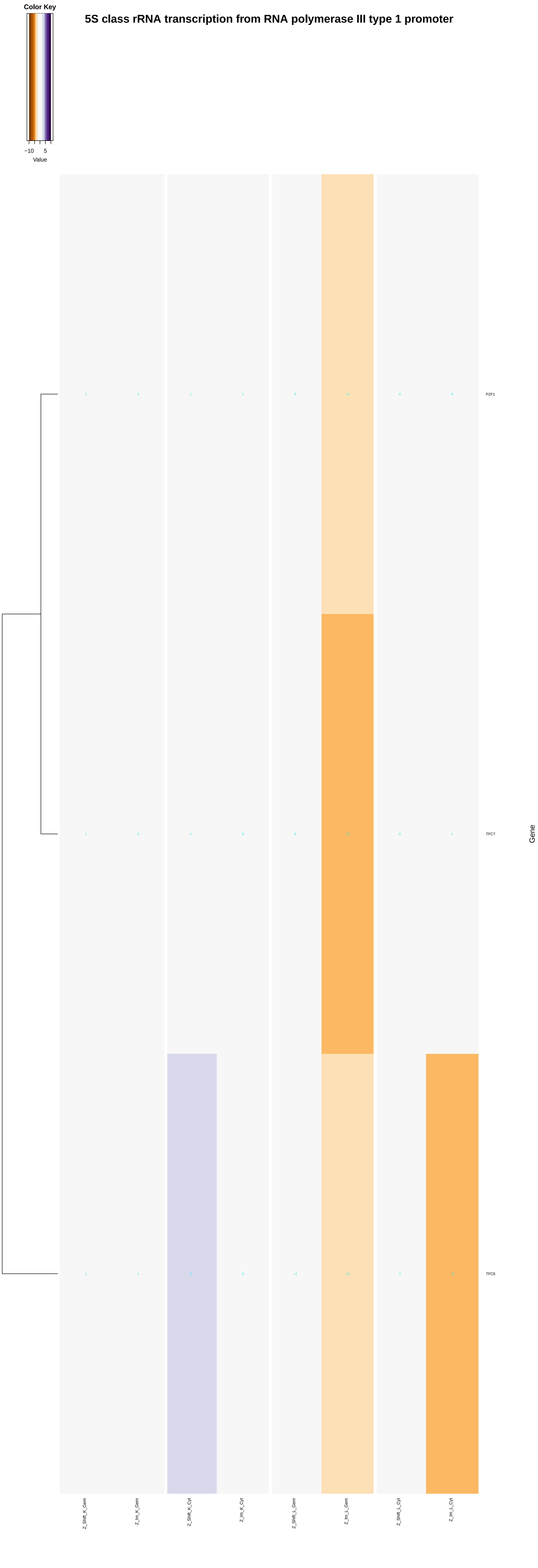
5S class rRNA transcription from RNA polymerase III type 1 promoter
Color Key
−10
5
Value
PZF1
TFC7
TFC6
Gene
Z_Shift_K_Gem
Z_Im_K_Gem
Z_Shift_K_Cyt
Z_Im_K_Cyt
Z_Shift_L_Gem
Z_Im_L_Gem
Z_Shift_L_Cyt
Z_Im_L_Cyt

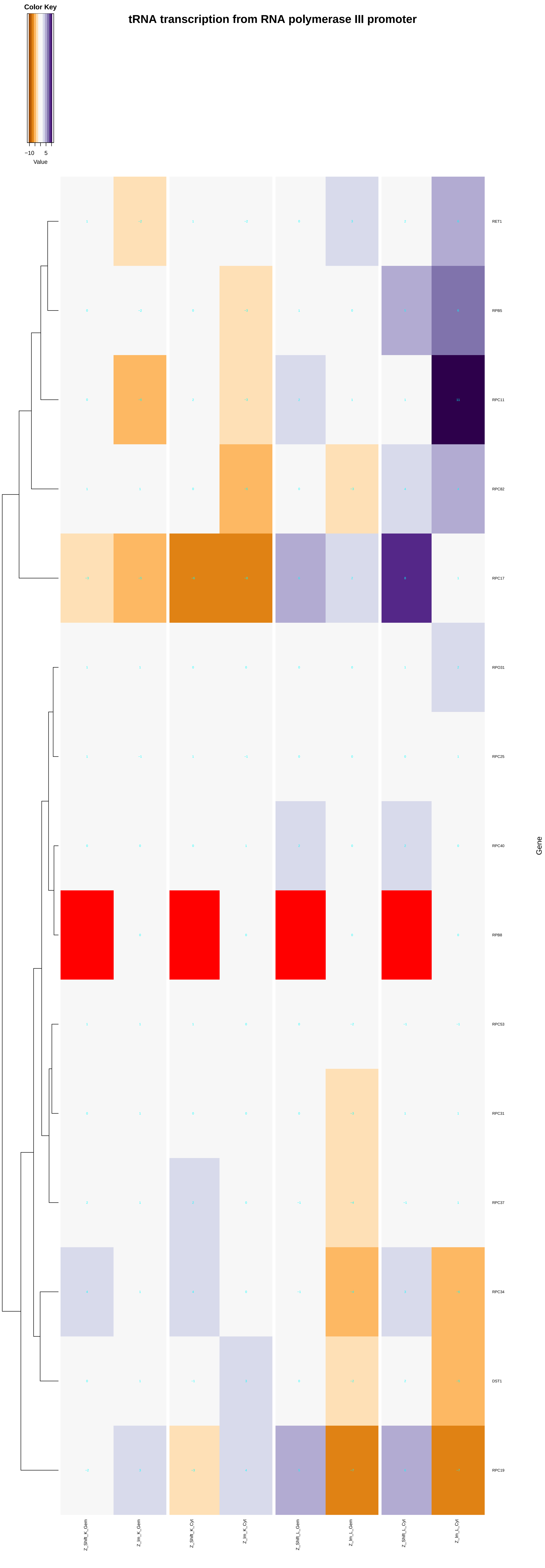

Color Key
−10
5
Value
tRNA transcription from RNA polymerase III promoter
RET1
RPB5
RPC11
RPC82
RPC17
RPO31
RPC25
RPC40
RPB8
RPC53
RPC31
RPC37
RPC34
DST1
RPC19
Gene
Z_Shift_K_Gem
Z_Im_K_Gem
Z_Shift_K_Cyt
Z_Im_K_Cyt
Z_Shift_L_Gem
Z_Im_L_Gem
Z_Shift_L_Cyt
Z_Im_L_Cyt

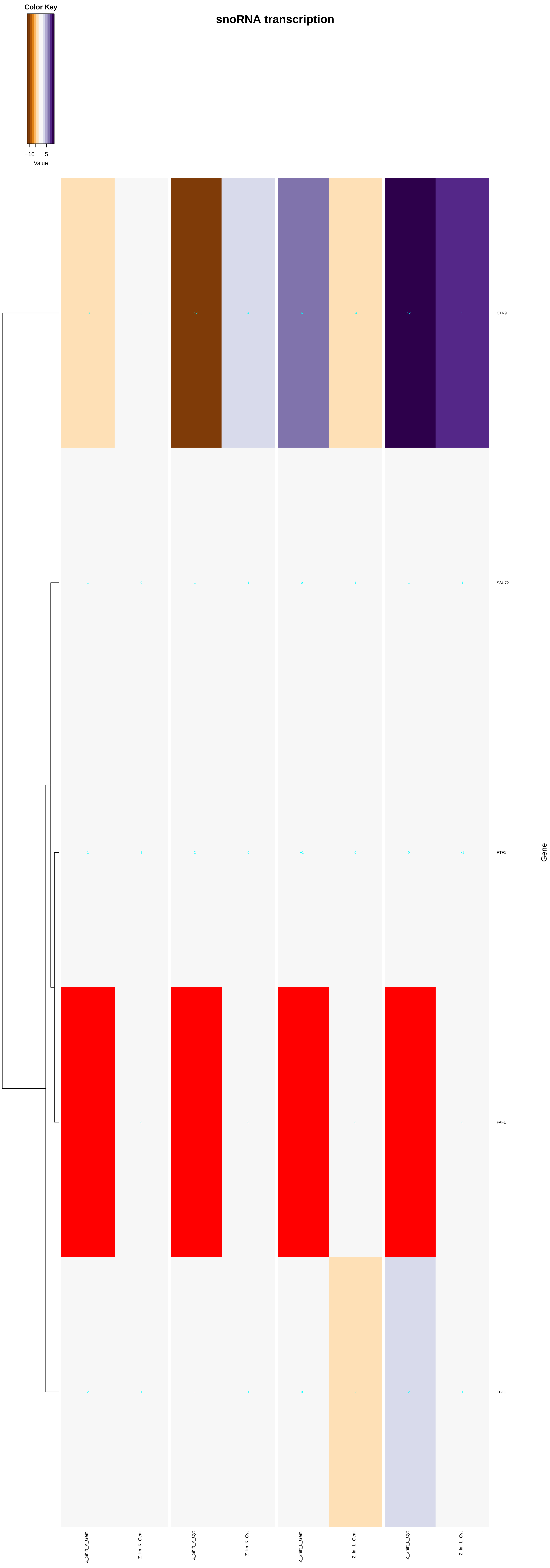
snoRNA transcription
Color Key
−10
5
Value
CTR9
SSU72
RTF1
PAF1
TBF1
Gene
Z_Shift_K_Gem
Z_Im_K_Gem
Z_Shift_K_Cyt
Z_Im_K_Cyt
Z_Shift_L_Gem
Z_Im_L_Gem
Z_Shift_L_Cyt
Z_Im_L_Cyt

rRNA transcription

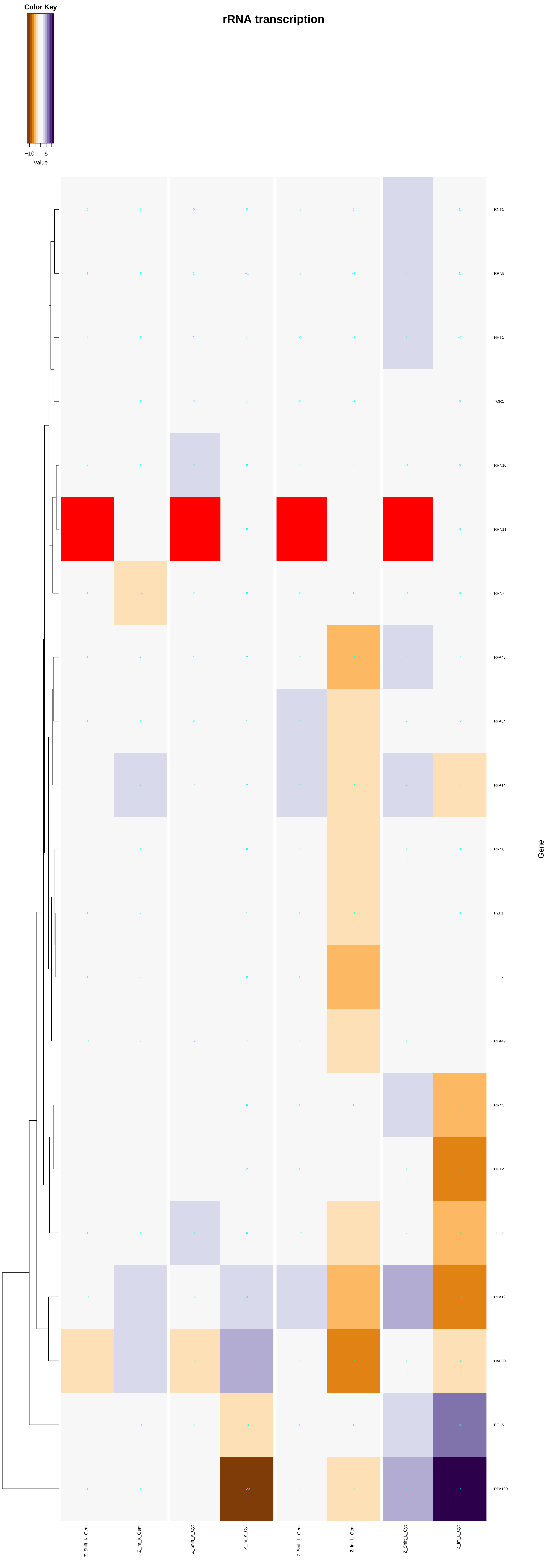

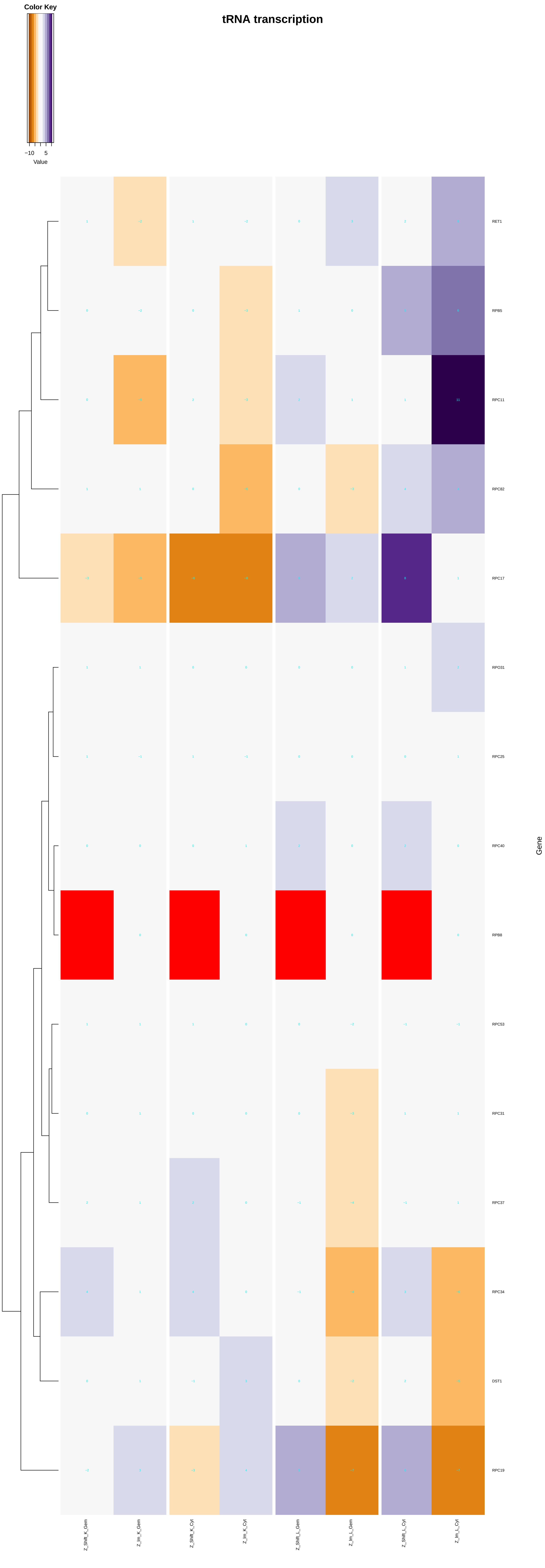
tRNA transcription
Color Key
−10
5
Value
RET1
RPB5
RPC11
RPC82
RPC17
RPO31
RPC25
RPC40
RPB8
RPC53
RPC31
RPC37
RPC34
DST1
RPC19
Gene
Z_Shift_K_Gem
Z_Im_K_Gem
Z_Shift_K_Cyt
Z_Im_K_Cyt
Z_Shift_L_Gem
Z_Im_L_Gem
Z_Shift_L_Cyt
Z_Im_L_Cyt
